# Adaptation of an electronic dashboard to monitor HIV viral load testing in Côte d’Ivoire

**DOI:** 10.4102/ajlm.v10i1.1284

**Published:** 2021-05-17

**Authors:** Mary Kirk, Paul H. Assoa, Casey Iiams-Hauser, Yves-Rolland Kouabenan, Jennifer Antilla, Caleb Steele-Lane, Greg Rossum, Pascal Komena, Patricia Sadate Ngatchou, Nadine Abiola, Alain Kouakou, Adama Pongathie, Jean B. Koffi, Christiane Adje, Lucy A. Perrone

**Affiliations:** 1 I-TECH, Department of Global Health, Schools of Public Health and Medicine, University of Washington, Seattle, Washington, United States; 2 International Training and Education Center for Health (I-TECH), Abidjan, Côte d’Ivoire; 3 Department of Global Health, University of Washington, Seattle, Washington, United States; 4 Direction de l’Informatique et de l’Information Sanitaire (DIIS), Ministry of Health, Abidjan, Côte d’Ivoire; 5 Division of Global HIV and Tuberculosis, Centers for Disease Control and Prevention, Abidjan, Côte d’Ivoire

**Keywords:** HIV viral load, electronic dashboard, patient monitoring, laboratory information system, Côte d’Ivoire

## Abstract

**Background:**

The Ministère de le Santé et de l’Hygiène Publique in Côte d’Ivoire and the international community have invested in health information systems in Côte d’Ivoire since 2009, including electronic laboratory information systems. These systems have been implemented in more than 80 laboratories to date and capture all test results produced from these laboratories, including HIV viral load (VL) testing. In 2018 the national HIV programme in Côte d’Ivoire requested international support to develop real-time tools such as dashboards to aggregate and display test-specific data such as HIV VL testing to support the country’s programmatic response to HIV.

**Intervention:**

The VL dashboard was adapted in 2018 using source software code obtained from the Kenyan Ministry of Health and modified for the Ivorian context. The dashboard enables users to assess relevant clinical data from all Ivoirians living with HIV who undergo VL testing through dashboard data visualisations, including the number of VL tests, kinds of samples tested, and VL levels stratified by demographics and geographic location.

**Lessons learnt:**

The VL dashboard enables rapid analysis of VL testing data from across the country and enables the national HIV programme, donors and partners to respond rapidly to issues pertaining to access, turn-around times and others.

**Recommendations:**

Adapting existing open-source software is an effective and efficient way to implement transformative tools such as dashboards. The VL dashboard will likely be an essential tool for Côte d’Ivoire to meet the United Nations Programme on HIV/AIDS 90-90-90 targets.

## Background

As of 2018, Côte d’Ivoire had approximately 460 000 people living with HIV^1^ and an estimated HIV prevalence rate of 2.6%,^1^ the highest in West Africa.^2^ To address unmet goals for the Joint United Nations programme on AIDS/HIV 90-90-90 targets, Côte d’Ivoire adopted the *Test and Start* approach in 2015, a proven cost-effective and lifesaving intervention.^3^ Côte d’Ivoire relies on viral load (VL) testing as the gold standard for clinical monitoring of HIV, yet VL testing remains limited in Côte d’Ivoire despite 17 laboratories having molecular testing capability. Côte d’Ivoire is working to scale up access to VL testing and optimise how test results are utilised for effective anti-retroviral therapy monitoring in pateints.^4^ The national HIV programme and the healthcare workforce in Côte d’Ivoire have been advocating with the international donor community for effective, user-friendly health information systems and monitoring tools.^5^ In response to this request, the United States Centers for Disease Control and Prevention engaged the International Training and Education Center for Health (I-TECH) at the University of Washington in 2009 to support the Ministère de le Santé et de l’Hygiene Publique and the national HIV programme (Programme Nationale de Lutte contre le Sida [PNLS]) to improve the quality of care of people living with HIV by strengthening electronic laboratory information systems as part of the national health information system architecture.

OpenELIS (electronic laboratory information systems) (OE) is an open-source electronic laboratory information system that tracks laboratory processes and outputs and was created in the United States for public health laboratories as a software and business process framework.^6^ Starting in 2009, OE was adapted for Côte d’Ivoire with I-TECH’s support and 82 laboratories to date are implementing the system across the nation. Although implementation of OE continues to expand and efforts are underway to interlink individual operating sites and their data through a centrally located consolidated server, obtaining aggregated HIV-related testing data from all sites remains challenging. In 2018 the Ministère de le Santé et de l’Hygiene Publique requested support to develop and deploy a dashboard solution for HIV VL testing.

Dashboards are increasingly used in clinic-based interventions^7^ as well as in population-based disease assessments.^8^ These dashboards can empower stakeholders to make decisions with greater efficiency over time.^9^ One primary function of dashboards is displaying data so that stakeholders can analyse key metrics to monitor progress to targets, identify challenges and mobilise to improve service quality.^10^ Dashboards have been used for reporting purposes in HIV programme monitoring efforts. In Uganda, Option B+ usage has been documented since 2013 via a nationwide weekly reporting system using short message service technology.^11^ Kenya also uses a dashboard to support the national review of VL and infant virologic test programme data through the integration of open-source laboratory information systems with cloud-based servers.^12^ Namibia developed a Pharmaceutical Management Information Dashboard that interlinks four pharmaceutical information tools to serve as a platform for analysis and dissemination to improve anti-retroviral therapy delivery.^13^ Here we describe the development of the VL dashboard for Côte d’Ivoire which is connected to the OE system and aggregates site-level OE data that can be viewed on a publicly available website. These visualisations permit the rapid examination of HIV-related testing data in Côte d’Ivoire, including spatial and temporal attributes of VL testing coverage, and support timely programme policymaking.

## Description of the intervention

### Ethical considerations

Ethical clearance for this project was given by the University of Washington, United States Centers for Disease Control and Prevention and Ivorian Institutional Review Board. All de-identified data are publicly available on the dashboard website.

### Dashboard development

In October 2017, I-TECH coordinated with the United States Agency for International Development, Clinton Health Access Initiative, and the Kenyan Ministry of Health to access the software source code for the Kenyan VL dashboard (https://viralload.nascop.org/).^14^ Using this software source code as a starting point, the Ivorian VL dashboard was developed to interface with the OE system in Côte d’Ivoire and designed to extract and aggregate VL test-specific data.

Populating the dashboard with data initially occurred through the manual extraction of a comma-separated value file from each OE system at each laboratory. Trained staff then exported this file to data quality analysts through remote connection access or email. A data validation check was then conducted to ensure that data were not missing or inaccurate, otherwise it was sent back to the laboratories for correction in the OE system. Once individual laboratory data were validated and complete, these cleaned data were then manually uploaded to the VL dashboard (note that a direct link from the OE systems to the VL dashboard through a national consolidated server is in its pilot phase as of September 2020, and this automation will remove the need for manual data management processes). The visualisations that populate the VL dashboard were adapted from the Kenyan dashboard to the Ivorian context, including geography, language, and data sources.

### Dashboard features

**FIGURE 1:**
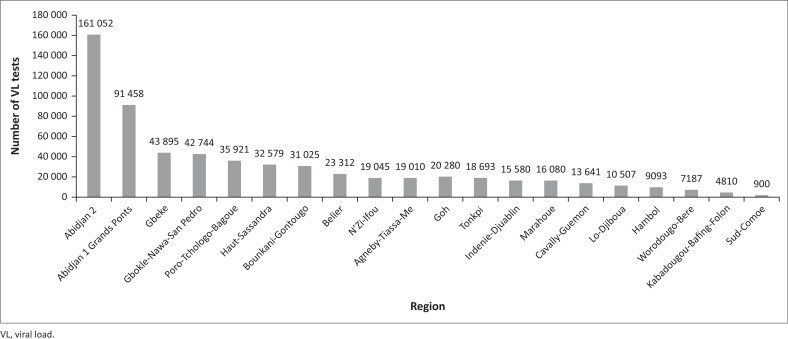
Number of viral load tests in Côte d’Ivoire by region, October – December 2019. The 20 regions of the country are covered by 17 public laboratories that are capable of molecular testing for HIV viral load. The capital Abidjan is divided into two jurisdictions based on geospatial parameters with the most people living with HIV located in Abidjan 2. This graph was produced directly from the viral load dashboard.

The VL dashboard was designed to display a range of data visualisations that can be sorted by month, year and region (Figure 1). The primary source data for ‘Trends by Testing Sample’ (test sample type by month) can be exported to Microsoft Excel (Microsoft Corp. Redmond, Washington, United States); the remaining data visualisations are available as graphic downloads. Test sample type (dried blood spot or ethylenediaminetetraacetic acid plasma), demographic data (age, sex, and region), test results (≥/< 1000 copies, invalid, or undetectable), and clinical justification for the VL test can be visualised on the VL dashboard. The turn-around times of the clinical VL samples can also be visualised, with the graphic displaying times from collection of the sample to reception at a laboratory, reception to processing, and processing to sample validation. Data submitted by each laboratory are available by regionally filtering data, with test numbers by results shown on the ‘Region Sites Outcomes’ graph on the dashboard. A ‘Visitor Counter’ tracking feature was added to the dashboard in January 2020, showing the total number of visits, number of visits to a specific page of the dashboard, and the number of people online.

## Lessons learnt

**FIGURE 2:**
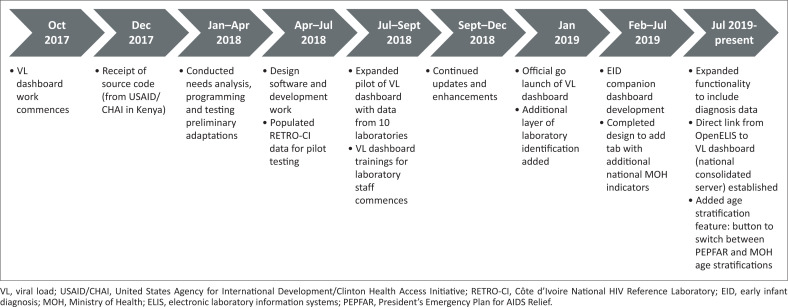
Timeline of viral load dashboard development. The development of the viral load dashboard began in October 2017 and a pilot version was completed in July 2018. Following adjustments to the software code the dashboard was finalised in January 2019 and is now populating data from all viral load testing laboratories.

**FIGURE 3:**
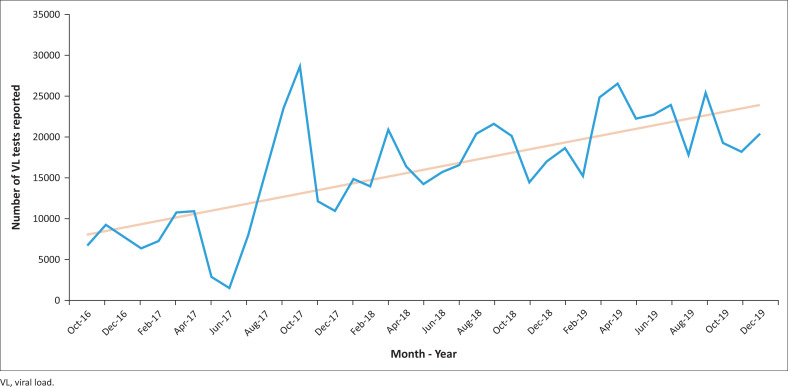
Number of viral load tests reported in Côte d’Ivoire, October 2016 – December 2019. The viral load dashboard captured all viral load testing data since its beginning in Côte d’Ivoire, October 2016. As the country committed to scaling up viral load testing access to people living with HIV, the number of total tests increased. The data represented above show this trend, which is expected to continue.

**FIGURE 4:**
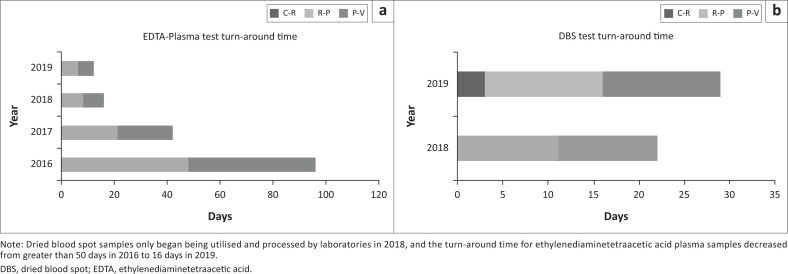
Viral load testing turn-around time by sample type and by year that each type of viral load test was used. (a) Ethylenediaminetetraacetic acid plasma 2016–2019; (b) Dried blood spots 2018–2019, in Côte d’Ivoire. Turn-around time is further disaggregated by collection to reception (C-R), reception to processing (R-P), and processing to validation (P-V).

The VL dashboard was completed in June 2018 (Figure 2). In July 2018, initial pilot data from the dashboard were presented to Ministère de le Santé et de l’Hygiene Publique and donor stakeholders at a technical working group meeting for final dashboard approval. The website became publicly available in July 2018 (https://chargevirale.openelisci.org/vl_dashboard/), with an initial 10 VL testing laboratories successfully transmitting data to the dashboard via OE. The remaining seven laboratories started data sharing from January 2019, and retrospective data from October 2016–2018 were obtained, cleaned, and uploaded into the dashboard. The VL dashboard successfully reported data for all people living with HIV who received VL testing services in Côte d’Ivoire, with each unique test event recorded in the dashboard. Data from October 2016 to December 2019 indicated that a total of 622 500 samples were tested for VL, of which 452 896 (72.8%) samples were from women and 169 604 (27.2%) from men. Regional disaggregation of the data accounts for 616 812 VL tests, with the remaining 5688 tests having lost location traceability and are currently under investigation. Of the 616 812 traceable VL tests, the two Abidjan catchment areas accounted for 40.6% (*n* = 252 510) of the tests in the dashboard. Of the VL tests reported in the dashboard, 89.6% were from patients aged 25 years or older. Since the dashboard became active, the sample processing rate has increased, reflecting increased VL testing access and scale-up and increased test demand after national prioritisation in 2016 (Figure 3). Data from October 2016 to December 2019 indicate that 56.9% (354 184 tests) of people living with HIV receiving anti-retroviral therapy have viral suppression. People living with HIV aged 25 years or older had the highest suppression rate (79%), and paediatric and adolescent patients (aged 0–19 years) had the lowest rates. Paediatric (age < 10 years) viral suppression rates ranged from 47.6% to 56.9%. Adolescent (age 10–19 years) suppression rates ranged from 53.9% to 55.2%. Although the dashboard indicated that the national average turn-around time for ethylenediaminetetraacetic acid plasma samples decreased from more than 50 days to 16 days (Figure 4), this does not yet meet the national target of under 14 days. Viral load results obtained from tests performed on dried blood spots were introduced in 2018.

The adaptation, development and launch of the VL dashboard in Côte d’Ivoire is a collaborative success story and a milestone achievement for the national HIV programme and for international donors such as the United States President’s Emergency Plan for AIDS Relief. The information available through the dashboard will help accelerate decision-making and programmatic response time,^11^ especially the ability to monitor key subpopulations. For example, paediatric and adolescent viral suppression rates are observably lower (53.4%) than adults aged 25 years or older (79.8%), and the dashboard is being used to monitor this population subset and track their clinical outcomes. It is noted that these VL suppression rates by age groups mirror trends among paediatric, adolescent, and adult patients in other resource-limited countries.^15^ This kind of information is critical to inform programmes and initiatives like the Determined, Resilient, Empowered, AIDS-free, Mentored, and Safe, which serves adolescent girls and young women. In another example of the utility of the dashboard, although only 23.9% of all people living with HIV in Côte d’Ivoire^1^ live in the capital region of Abidjan, dashboard data from October 2016 through December 2019 show that a large proportion of VL samples come from this region (*n* = 252 510 tests, 40.6%; Figure 2). This finding suggests that more people from Abidjan are receiving VL tests compared with other provinces and these data are now informing programmatic prioritisation. Organisations in Côte d’Ivoire are now rapidly utilising VL dashboard information in programmatic and technical working group meetings. The PNLS uses the data and visualisations from the dashboard in monthly reviews with implementing partners and other ministerial departments. The data from the VL dashboard is trusted in the PNLS’s decision-making processes.

One of the key success factors in the development of the dashboard was the collaborative nature of the work in each step of the process: from planning to design and implementation. The willingness of the developers of the Kenyan dashboard to share the source code immensely benefited Côte d’Ivoire, as it shortened product launch time, reduced potential costs and prevented duplication of efforts to implement a dashboard for Ivorian VL testing services. In the adaptation phase, I-TECH worked closely with the Direction de l’Informatique et l’Information Sanitaire, the PNLS, and the Côte d’Ivoire National HIV Reference Laboratory in monthly technical working group meetings. The technical working group first reviewed the data available in OE and mapped the data to the indicator calculations. After the VL dashboard was launched, these high-functioning technical working groups provided a forum to address challenges and improve the dashboard and included representatives from the Direction de l’Informatique et l’Information Sanitaire, PNLS, Laboratoire National de la Santé Public, and implementing partners. This ensured that all stakeholders were involved in the process.

The VL dashboard has been a helpful tool in monitoring HIV trends towards meeting the ‘third 90’ United Nations Programme on HIV/AIDS goal and additional developments are underway to enhance features and improve clinical utilisation. The VL dashboard is also a model for the new early infant diagnosis dashboard launched in May 2020.^16^ Completion of the consolidated national server, which is in its pilot phase since September 2020, will allow for rapid data importation from OE to the VL dashboard. Plans are also underway to transfer ownership of the dashboard’s internet protocol address, currently owned by the University of Washington, to the Ministère de le Santé et de l’Hygiene Publique-Direction de l’Informatique et l’Information Sanitaire.

## Recommendations

Dashboards are helpful tools for public health programmes. The VL dashboard in Côte d’Ivoire is poised to be a transformational tool that improves the national response to HIV by displaying key data including the number of VL tests, kinds of samples tested, and VL levels stratified by demographics and geographic location. This dashboard enables rapid analysis of VL testing data from testing points across the country and will enable the PNLS and other implementing partners to respond rapidly to VL testing access, delayed turn-around time, and data quality issues. The VL dashboard will help Côte d’Ivoire scale up VL monitoring to help meet the United Nations Programme on HIV/AIDS 90-90-90 targets.
